# Divergence and efficiency optimization in polarization-controlled two-color high-harmonic generation

**DOI:** 10.1038/s41598-021-03657-2

**Published:** 2021-12-20

**Authors:** Sylvianne D. C. Roscam Abbing, Filippo Campi, Alexandra Zeltsi, Peter Smorenburg, Peter M. Kraus

**Affiliations:** 1 Advanced Research Center for Nanolithography, Science Park 106, 1098 XG Amsterdam, The Netherlands; 2 ASML Research, ASML Netherlands B.V., 5504 DR Veldhoven, The Netherlands; 3 Department of Physics and Astronomy, and LaserLaB, Vrije Universiteit, De Boelelaan 1105, 1081 HV Amsterdam, The Netherlands

**Keywords:** High-harmonic generation, X-rays, Nonlinear optics

## Abstract

Improving the brightness of high-harmonic generation (HHG) sources is one of the major goals for next-generation ultrafast, imaging and metrology applications in the extreme-ultraviolet spectrum. Previous research efforts have demonstrated a plethora of techniques to increase the conversion efficiency of HHG. However, few studies so far have addressed how to simultaneously minimize the divergence and improve focusability, which all contribute to an increased brightness of the source. Here, we investigate how to improve both photon yield and divergence, which is directly linked to focusability, when adding the second harmonic to the fundamental driving field. We study the effects of the relative polarization in two-color HHG and compare the results to a one-color configuration. In a perpendicular two-color field, the relative phase between the two colors can be used to suppress or enhance recombination of either the long or the short trajectories. This allows to exert control over the divergence of the harmonics. In a parallel two-color field, the ionization rate is modified through the two-color phase, which selects trajectories during the ionization step. This enhances the total yield. We elaborate on the underlying mechanisms for parallel, perpendicular, and intermediate polarization angles, and confirm our experimental observations with simulations.

## Introduction

High-harmonic generation (HHG) is the cornerstone of attosecond science^1–6^ and ultrafast (table-top) extreme-ultraviolet and soft-X-ray science^7^ ever since its discovery in 1987/1988^8,9^. Furthermore, applications in coherent diffractive imaging^10–15^, spectroscopy^16–20^ and metrology in industry^21–23^ benefit from the short emitted wavelengths and the associated potentially high spatial resolution. All these applications would, however, benefit tremendously from higher conversion efficiencies and better focusability of the generated pulses.

**Figure 1:**
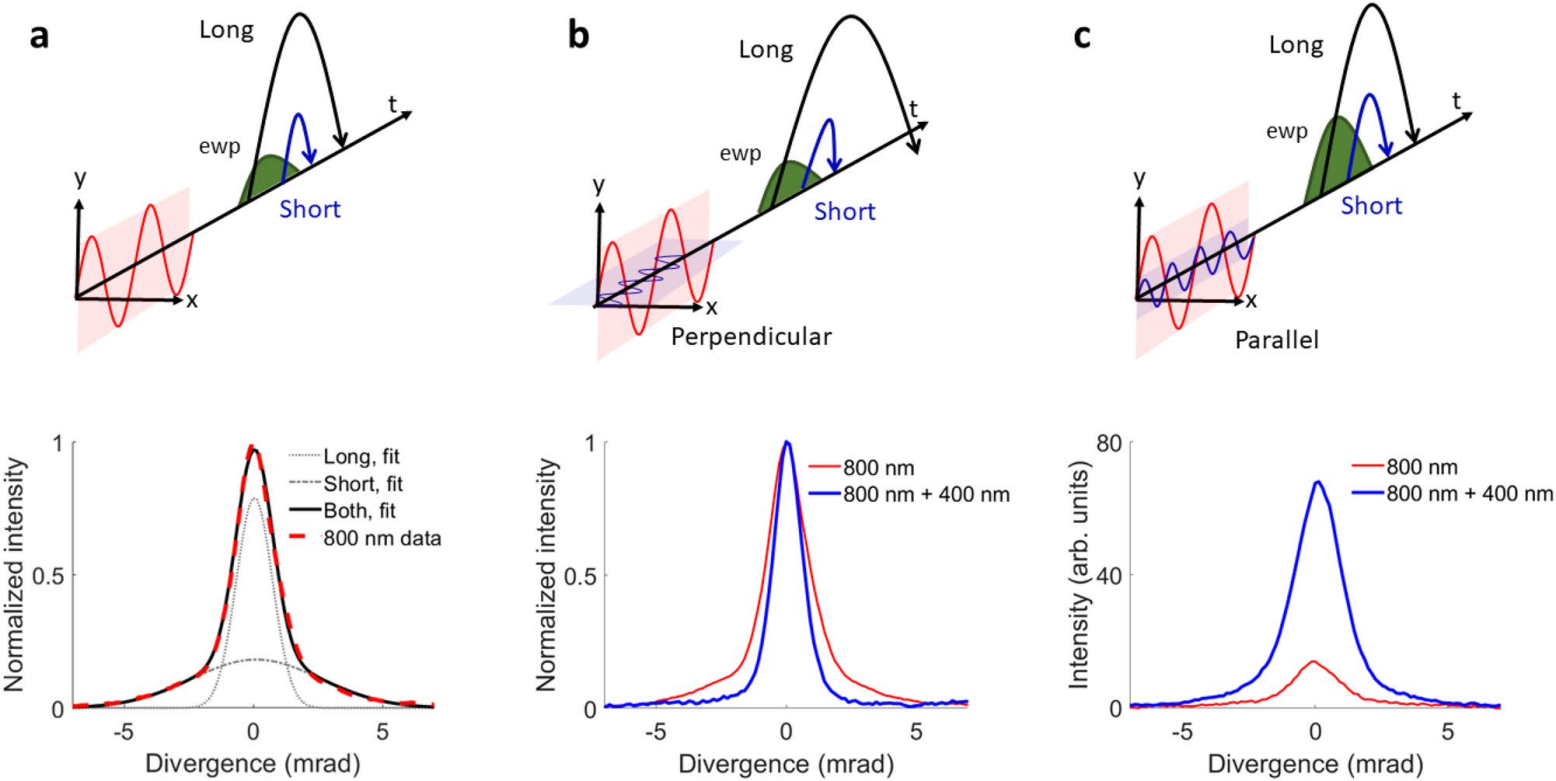
Two-color HHG for different relative polarizations. (**a**) HHG using 800 nm fs pulses. Both long and short trajectories, represented as an electron wave packet (ewp, green) at the instant of tunnel-ionization, contribute to the generation (top panel). The spectrally integrated data (dashed red line) follows a double-Gaussian intensity profile (black line) as function of emission angle (divergence) in the far-field (bottom panel). The broader Gaussian envelope (dashed-dotted line) corresponds to the long trajectory contribution, whereas the narrower Gaussian envelope (dotted line) represents the short trajectory contribution. (**b**) HHG using 800 nm pulses and perpendicular polarized 400 nm pulses. Due to the lateral component of the electric field, long trajectories can be made less likely to recombine (top panel, represented by the black arrow ending up off-axis) for an optimized two-color delay. This leads to a beam profile with suppressed signal in the wings, which is closer to a mono-Gaussian beam profile than in one-color HHG (bottom panel, showing normalized beam profiles for comparing the divergence). (**c**) HHG using 800 nm pulses and parallel polarized 400 nm pulses. Due to an increased ionization rate for electrons (represented by the higher amplitude of the electron wave packet) (top panel), the total yield can be enhanced for optimized two-color delays, compared to the monochromatic case (bottom panel).

The origins of divergence and better focusability can be understood by the mechanism of HHG. Irradiation of a gas with a strong laser field leads to ionization of electrons from the parent ions. The electrons are accelerated in the continuum and accumulate an intensity-dependent phase during propagation, before they recombine with the parent ion, leading to the generation of XUV photons^24–26^. Each generated energy is the superposition of the contributions of two electron paths through the continuum, the short and the long trajectories, which lead to a different phase upon recombination, leading to emission of two differently curved wavefronts. This effect leads to a double-Gaussian profile of the harmonic beam in the far-field^27–31^, as shown in Fig. 1a. The double profile is a manifestation of the two trajectories having different virtual foci, which cannot be reimaged to the same plane with a subsequent focusing element, thus diminishing the overall focusability of the beam^27,32,33^. Thus the far-field divergence and its deviation from a mono-Gaussian profile is a signature of the focusability of the beam.

In order to increase the brightness of the HHG beam, control over the short and long trajectories is crucial^34^, as selection of one of the two species improves the focusability. Previous work^34–41^ had already pointed out that two-color HHG can improve the conversion efficiency by shaping the driving laser field, thus controlling both ionization and the recollision of continuum electrons that lead to HHG. In an earlier work^27^, we demonstrated the use of a perpendicular two-color field to manipulate the generation mechanism and thus control the divergence of HHG. In particular, we were able to enhance the emission of either the short or the long trajectories, depending on the relative two-color phase between the fields. The double-Gaussian beam profile can be improved significantly by selecting the short trajectories over the long trajectories, as shown in Fig. 1b. Due to the addition of a perpendicular second harmonic (SH) field during generation, the electrons receive a lateral momentum component, preventing the recombination of either the long or the short trajectories, depending on the two-color phase. This configuration also leads to an overall increase of the yield, compared to a one-color generation scheme, due to an enhanced ionization of the medium (Argon). This reshaping of the field to enhance ionization of a narrow trajectory window is even more efficient in two-color fields with parallel polarization^42^.

More recently, it has been realized that linearly and cross-polarized two-color fields with angles between fundamental and second harmonic that deviate from 0 or 90° can generate elliptically and circularly polarized HHG^43–46^. This important property motivates a systematic investigation how yield and divergence are affected in cross-polarized two-color HHG.

We therefore set out to systematically investigate both experimentally and theoretically which relative polarization configuration in two-color HHG in Argon is best suited to simultaneously optimize divergence, focusability, and overall photon yield. We compare the two-color HHG spectra with a one-color configuration for the spectral range of harmonic 13 to harmonic 25. In addition to a perpendicular two-color configuration^27^, we also generate harmonics in parallel two-color fields, and several intermediate polarization angles. The use of a parallel two-color field specifically consisting of a fundamental and its SH shows the highest increase of the yield of the harmonics for optimized two-color phases, by at least a factor of 5, as illustrated in Fig. 1c. By comparing experiments and simulations, we show that HHG in a two-color field can favor the emission of plateau harmonics through trajectory reshaping and selection and enhance the overall yield for specific two-color phases. In perpendicularly polarized two-color fields, the second color additionally deflects certain continuum electron trajectories without significantly reshaping their emission times and excursion amplitudes, which favorably narrows the divergence, but does not increase the yield as much as for parallel polarized two-color fields. The simulations, which included the single-atom response of the atoms to the intense laser pulses as well as macroscopic phase matching, allow to quantitatively simulate beam profiles in two-color HHG. The simulations fully agree with the experiments and reveal that the single-atom response alone qualitatively explains the yield enhancement and divergence minimization for parallel and perpendicularly polarized two-color fields, respectively.

## Results and discussion

### Simulated yield and trajectory contributions of two-color HHG

We start by simulating our two-color HHG experiments in order to provide physical insight into the divergence control for perpendicularly polarized two-color fields (Fig. 1b and Ref.^27^), and to establish concepts how these results translate to different polarizations.

**Figure 2:**
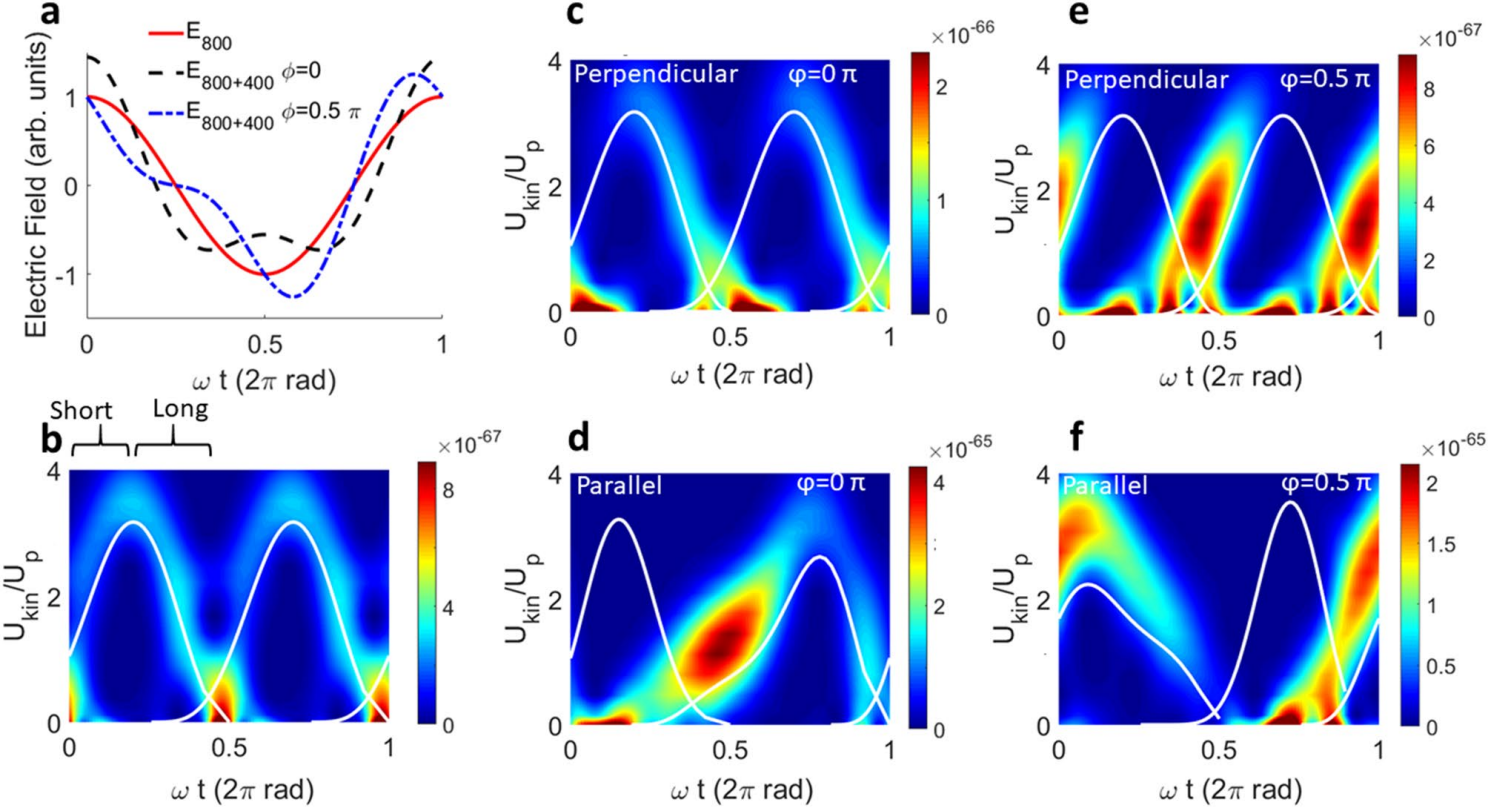
Time and frequency resolved emission. The color scale represents the squared dipole moment in $\text {C}^2\text {m}^2$. The white lines are the semi-classical calculation of the frequency versus the recombination time. (**a**) Electric field for one and two colors, for two different two color phases. (**b**) For 800 nm only. Long and short trajectories are labeled in the first half-cycle emission (not labeled but clearly visible in the second half cycle). (**c**) Two-color phase of 0 rad, perpendicular configuration. (**d**) Two-color phase of $0.5\pi$ rad, perpendicular configuration. (**e**) Two-color phase of 0 rad, parallel configuration. (**f**) Two-color phase of $0.5\pi$ rad, parallel configuration.

The HHG process is simulated as a single-atom response^26,47^ in Fig. 2, and subsequently generalized to a realistic multi-atom response by propagation computations^48^ later in this manuscript. Figure 2 shows time-frequency analyses of a single-atom simulation in a single cycle of the fundamental electric field (Fig. 2a) for one-color (800 nm) HHG (Fig. 2b), perpendicular polarized two-color (800 nm + 400 nm) HHG (Fig. 2c,e) and parallel polarized two-color HHG (Fig. 2d,f) for two different phases of the two-color field, respectively. The combined field is defined as $E(t)=E_{\omega }\cos (\omega t) + E_{2\omega }\cos ( 2 \omega t + \phi )$, with $\omega$ the frequency of the fundamental field, and $\phi$ is the relative two-color phase between the fundamental and SH, as displayed for parallel polarizations in Fig. 2a. The return kinetic energy of the electrons is shown on the vertical axis in Fig. 2b–f normalized to the ponderomotive energy of a free electron in the laser field. The horizontal axis shows one full cycle of the fundamental laser field. The time dependent dipole moments are calculated in the strong-field approximation (SFA)^26,47^ as described in the “Methods” for more details. The color map represents the square of the dipole moment, which is proportional to the single-atom emission intensity and therefore also to the yield of the harmonics, if phase matching can be neglected. The white lines are the return kinetic energies, as calculated semi-classically according to the three-step model^24,25^.

In the case of a one-color driving field (Fig. 2b) the mapping of each harmonic energy onto the recombination times in each half cycle is clearly visible. The short trajectories are positively chirped (positive slope of the emission in the time-frequency plots) and the long trajectories are negatively chirped (negative slope), as indicated in Fig. 2b. We note that the SFA calculation agrees well with the semi-classical calculations. The emission intensity of the short and long trajectories is comparable, which is in agreement with the general observation of a double-Gaussian beam profile of the high-order harmonics.

With the addition of a perpendicular SH field, the relative ratio between the long and short trajectories is heavily modified by the relative phase of the two-color field, see Fig. 2c and e for two-color phases of 0 and $0.5\pi$ rad, respectively. The SH field imparts a lateral component to the momentum of the continuum electrons, thus preventing the recombination of either short (Fig. 2c, 0 rad) or long electron trajectories (Fig. 2e, $0.5\pi$ rad), depending on the relative two-color delay. In particular, the short trajectory selection for a relative phase of $0.5\pi$ is strongly enhanced, resulting in an overall increase of the total yield for this relative two-color phase. In the case of parallel two-color generation, the forces on the continuum electrons exerted by the two fields are aligned parallel and therefore trajectory selection by hindering recombination of electrons is not possible. Nevertheless, there is a clear difference in yield from the short or long trajectories as shown in Fig. 2d,f. This is because the two-color phase will act as gate for certain ionization times in the parallel case, leading to half-cycle emission with very different cutoff energies. For a relative two-color phase of 0 rad (black dashed line in Fig. 2a), the increased total electric field leads to enhanced ionization close to an absolute phase of the combined field of $\omega t = 0$ (x-axis in Fig. 2a)^49^. Ionization of electrons in this window will lead to emission of lower-energy photons, i.e. to a lower half-cycle cutoff than in the one-color case, because the absolute total electric field at the instant of recombination is lowered for subsequent absolute phases $\omega t$ of the combined fields.

The strong difference in half-cycle cutoffs becomes even more extreme for a relative two-color phase of $0.5\pi$ rad (Fig. 2f), and the same argumentation holds. The two half cycles lead to strongly different emission cutoffs, with the lower one contributing the highest yield.

Overall the simulations suggest stronger trajectory suppression and hence larger control over the divergence for perpendicular polarizations on the one hand, but estimate on the other hand a higher emission intensity for the parallel configuration, compared to the monochromatic and the perpendicular two-color configuration. We thus set out to test this theoretical indication both through simulations and experiments, in order to find optimum polarization and two-color phase configurations that allow for simultaneous divergence and yield optimization. In addition, the predicted difference in half-cycle cutoffs can be exploited for an absolute phase and intensity calibration of the two-color field, as demonstrated in the next section.

### Absolute calibration of two-color fields in HHG

**Figure 3:**
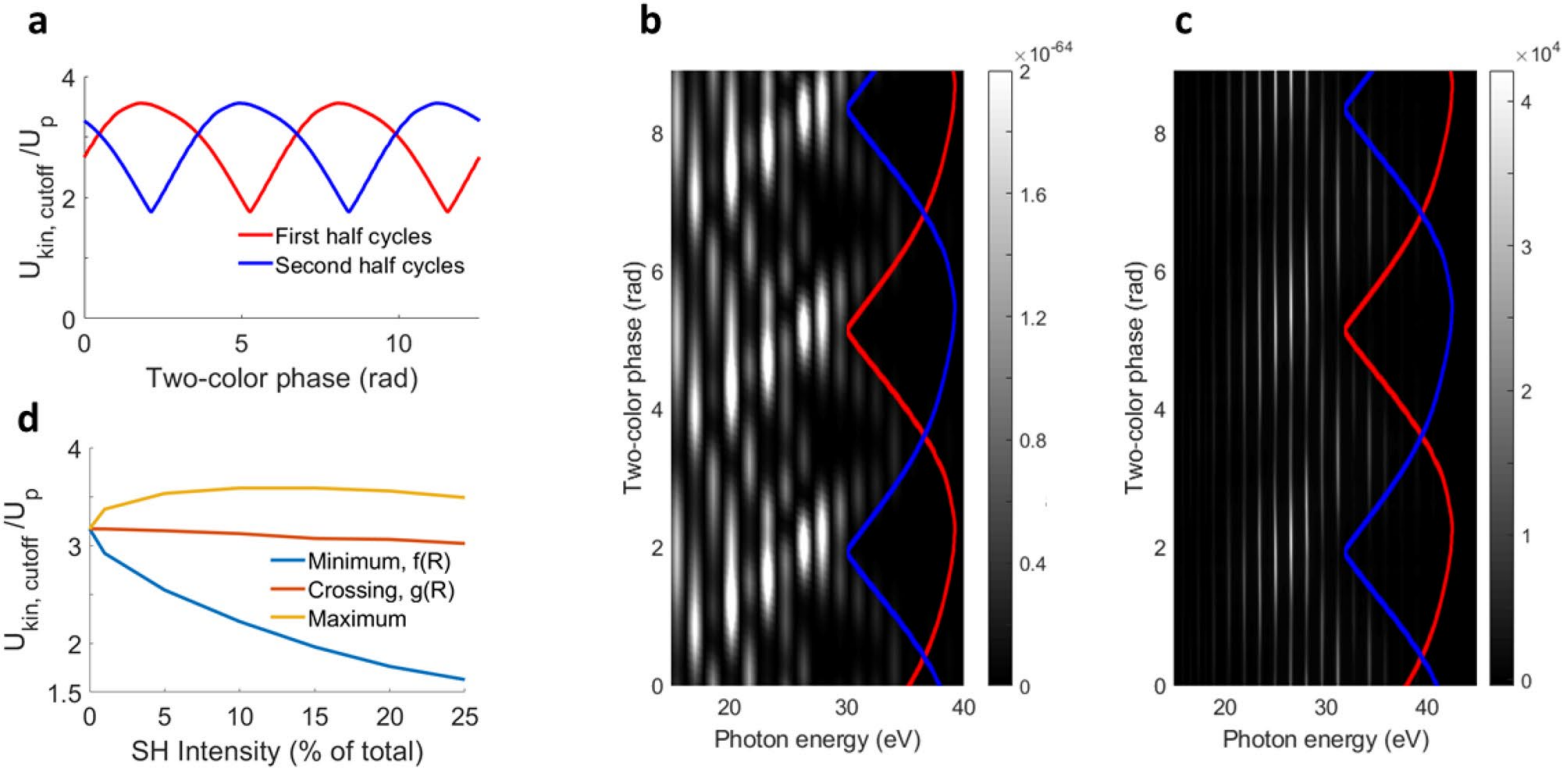
Cutoff modulation in parallel polarized two-color HHG. (**a**) Cutoff kinetic energy in adjacent half cycles, called first and second half cycle, as a function of two-color phase. Depending on the relative phase, each half cycle will have a different cutoff. (**b**) Simulated two-color HHG spectra as a function of two-color delay, showing how the cutoff modulates depending on the two-color phase delay. (**c**) Experimental HHG spectra as function of two-color phase, showing the same cutoff modulation as suggested in the simulations in (**b**). (**d**) Change of cutoff energy as a function of SH intensity. See text for details on the plotted functions.

We now turn to exploiting the strong cutoff modulations as a function of the relative two-color phase in parallel polarized two-color fields for calibrating the absolute two-color phase and absolute intensities of fundamental and SH. The simulations in Fig. 2d,f suggest that adjacent half cycles will gain different cutoff energies, which we now test experimentally in Fig. 3.

The kinetic energy of the returning electron in the cutoff for two adjacent half cycles, as obtained by semi-classical calculations, is shown in Fig. 3a. For delays where the two half cycles have the same cutoff, the maximum kinetic energy is close to the cutoff energy in one-color HHG (3.17 Up). The simulated and measured HHG spectra as a function of two-color phase, as shown in Fig. 3b,c respectively, show an excellent match and reveal that the cutoff modulation has a large impact on the yield of the generated harmonic spectra.

If we overlay the modulation of the cutoff of adjacent half cycles from Fig. 3a with simulated and measured spectra (red and blue lines in Fig. 3b,c), we observe that the lower branch generates the most intense harmonics, in agreement with SFA calculations shown in Fig. 2d,f. Comparing the cutoff and intensity modulation between experiment and simulation now allows calibrating the absolute two-color phase, which was used throughout this article.

In addition, we can calibrate the absolute intensities of the two-color field, as the cutoff-energy change as a function of two-color phase depends on the relative intensity of the fundamental and SH. For that purpose, the minimum and maximum cutoff energies $\hbar \omega _1$ and $\hbar \omega _2$ are extracted from the experimental data in Fig 3c. We call the kinetic energy of the minimum cutoff from the experimental data normalized to the ponderomotive energy $f(R)=(\hbar \omega _1 -I_p)/U_p$ (blue line in Fig. 3d), with $I_p$ the ionization potential and $U_p$ the ponderomotive energy. The kinetic energy of the maximum cutoff from the experimental data is $g(R)=(\hbar \omega _2 - I_p)/U_p$ (red line in Fig. 3d).

While the minimum cutoff in the experimental data corresponds to the simulated minimum half-cycle cutoff (minimum of blue and red lines in Fig. 3a–c), the maximum cutoff *g*(*R*) that is visible in the experimental data corresponds to the crossing of maximum and minimum cutoff of the simulated data (crossing of red and blue line in Fig. 3a–c). The maximum cutoff as obtained by the semi-classical calculation (maximum of blue and red lines in Fig. 3a–c) is barely visible, neither in the SFA simulation (Fig. 3b) nor in the experimental data (Fig. 3c). By comparing the measured kinetic energy corresponding to the crossing point (red line in Fig. 3d) and the minimum cutoff (blue line in Fig. 3d) to the predicted values as a function of relative SH intensity, we can determine the SH intensity fraction, and from the cutoff crossing point (which does not vary much with SH intensity fraction) the absolute intensity can be determined. With this procedure we obtain a total peak intensity of $1.4\cdot 10^{14}$ $\text {W/cm}^2$, a peak intensity of $1.2\cdot 10^{14}$ $\text {W/cm}^2$ for the fundamental, and a peak intensity of $0.2\cdot 10^{14}$ $\text {W/cm}^2$ for the SH.

While determining the peak intensity is in principle possible by measuring pulse duration, focus size and pulse energy separately, we find that the procedure outlined here gives far more accurate results, as it also reveals if fundamental and SH are properly temporally synchronized and spatially overlapped in focus. The method proposed here furthermore directly relates to the intensities that are involved in HHG as opposed to deducing intensities from a number of separate measurements.

### Divergence and yield optimization in two-color HHG

We now analyze the yield and divergence optimization in two-color HHG in detail, and determine which relative polarization between SH and fundamental in the two-color field most favorably influences these beam properties.

**Figure 4:**
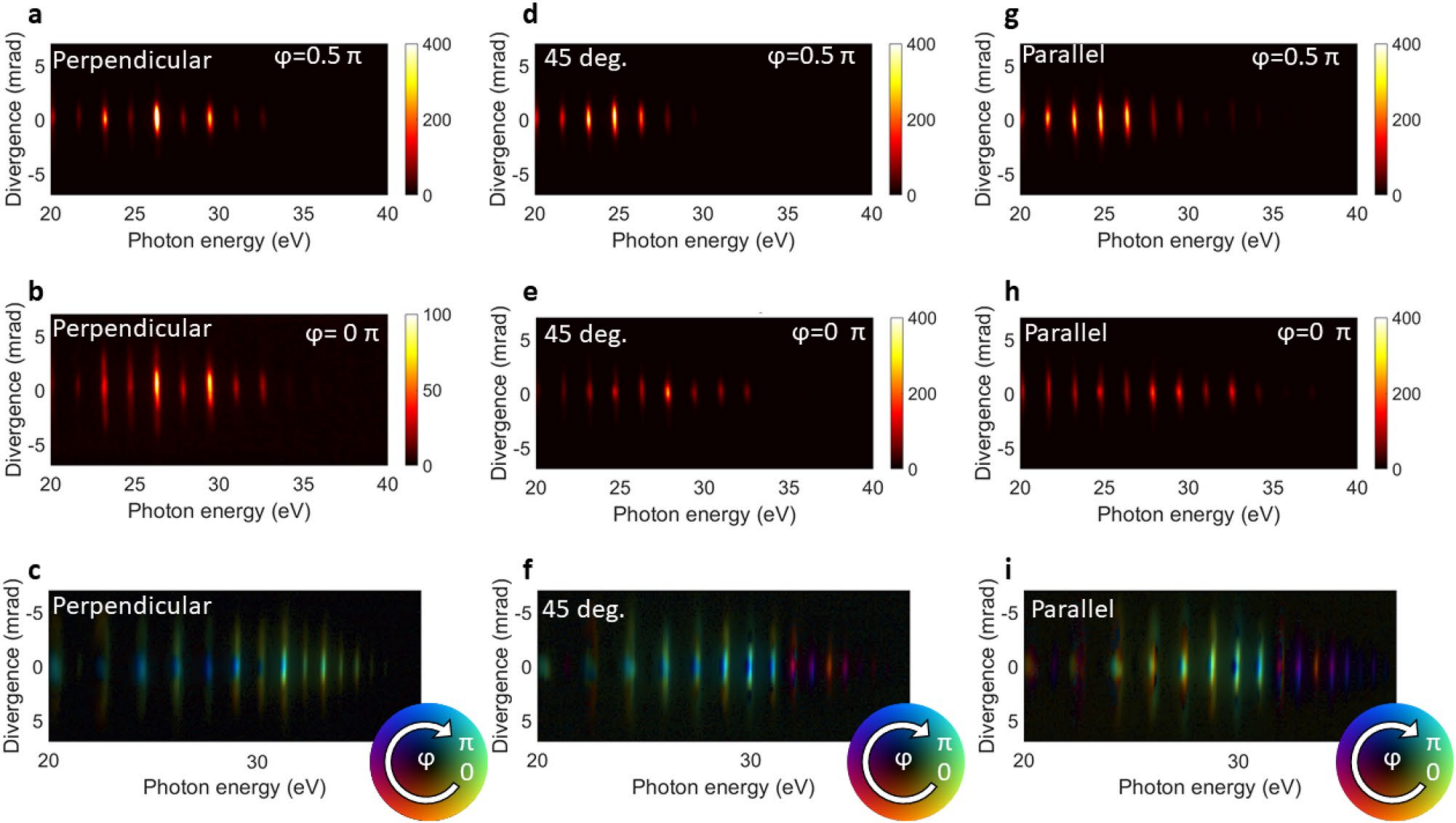
HHG spectra for different two-color phases and relative polarizations: (**a**,**b**) HHG spectra for perpendicular polarizations between SH and fundamental, for 0 and $0.5\pi$ rad, respectively. (**c**) Fourier map for perpendicular polarizations, indicating for each pixel at which two-color phase (color) the harmonic yield maximizes. Large divergence angles (yellow-orange) and smaller divergence angles (blue) indicate the existence of long and short trajectories, respectively. The brightness of each pixel indicates the amplitude of the harmonic signal. (**d**,**e**) HHG spectra for relative polarizations of 45°, for 0 and $0.5\pi$ rad, respectively (**f**) Fourier map for relative polarizations of 45°. (**g**,**h**) HHG spectra for parallel polarizations, for 0 and $0.5\pi$ rad, respectively. (**i**) Fourier map for parallel polarizations.

To this end, we have measured the divergence-resolved far-field high-harmonic spectra for three different relative polarizations between the two colors, and scanned the relative two-color phases, shown in Fig. 4. For a perpendicular configuration (Fig. 4a,b), both the shape and the yield of the spectrum change significantly for different two-color phases.

Figure 4a,b represent phase delays that generate a rather mono-Gaussian profile (Fig. 4a) and a more divergent profile (Fig. 4b), respectively. Whereas the generation in Fig. 4a is very centered, and most signal is contained in the smaller divergence angles, Fig. 4b shows a much larger distribution of signal across larger divergence angles. This demonstrates, in agreement with earlier findings^27^, that the divergence of the beam can be controlled by changing the relative two-color phase delay. This is directly related to the selection of short or long trajectories for different two-color phases as shown in our simulations in Fig. 2c,e. In addition, we also observe that the yield for the more mono-Gaussian profile is higher than for the more divergent spectrum.

We now apply a method we introduced in Ref.^27^, in order to compress yield and divergence modulations over an entire scan of the two-color phase delay into a single figure, shown in Fig. 4c. In this analysis, the far-field spectrum as function of two-color phase is Fourier transformed, and the most dominant frequency of this Fourier transform is shown in amplitude (brightness) and phase (color), producing a ’Fourier map’. Figure 4c thus displays at which two-color delay each pixel in the spectrum has the highest emission intensity. As long and short trajectories show a maximum emission intensity for different two-color phases, this method directly visualizes which parts of the divergence-resolved beam profile are dominated by long and short trajectories, respectively. More details on this analysis can be found in the “Methods” section and in Ref.^27^. There is a clear discrimination between the larger (yellow-orange) and smaller (blue) divergence angles for each harmonic order. This means that at the two-color delay related to the blue color, the harmonic signal that is coming from the smaller divergence angles is optimized, which we can relate to the emission of short trajectories. Oppositely, for the two-color delay related to the yellow-orange shade, the signal is maximized in the wings, caused by an increase of long trajectory emission. In addition, each harmonic order shows a slightly different color shade, denoting that each order has a different two-color phase at which either the short or the long trajectories optimize, reflecting the change in recombination times for different harmonics. For harmonic orders in the cutoff, long and short trajectories merge and thus there is no discrimination for emission between larger and smaller divergence angles, as can be seen in Fig. 4c from the absence of a phase (color) contrast for small and large divergences in cutoff harmonic orders.

The second column of spectra corresponds to measurements with a relative polarization of 45°. In Fig. 4d,e, we show spectra for 0 and $0.5\pi$ rad phase delay, that generate a high and a low total yield, respectively. There is a slight difference in divergence visible between the two spectra, but this is not as significant as for the perpendicular case in Fig. 4a,b. We notice that the cutoff changes by a few harmonic orders for the two different two-color phases in Fig. 4d,e. This modulation of the cutoff is especially visible in the analysis of the complete two-color scan in Fig. 4f, in which we see a color contrast for plateau and cutoff harmonics, which means their yield maximizes for different two-color phases. This is a direct result of the different half-cycle cutoffs of adjacent half cycles in (partially) parallel polarized two-color fields as shown in Fig. 3c, which lead to a strong cutoff modulation as a function of the two-color delay. Furthermore, we again observe a color difference between inner and outer divergences for each harmonic order, which is particularly pronounced for the plateau harmonics. This shows that a relative polarization angle of 45° provides an intermediate case, where the perpendicular SH component can still give a large enough lateral momentum to the electrons for trajectory selection, whereas the enhanced ionization through the parallel component of the SH field favorable enhances the yield. In addition, it has been reported^44–46^ that an intermediate polarization between two-color fields will lead to elliptically polarized harmonics. The manipulation of the electron trajectories by using two-color fields can lead to a classical angular momentum of the electrons with respect to the atom, due to a point of recombination of the electron which is at a distance from the nucleus. The additional asymmetry of adjacent half-cycles leads to an elliptical polarization.

In the right column, we analyze the results of two-color delay scans for parallel polarized two-color fields. Figure 4g shows the spectrum for a two-color phase of 0 rad, producing a high yield, and Fig. 4h shows the spectrum for $0.5\pi$, producing a low yield. Similar to the figures in the center column for 45° polarization in the two-color field and in agreement with the SFA simulations in Fig. 2, we see a cutoff extension in Fig. 4h, where the yield is lowest. The analysis of the complete two-color scan in Fig. 4i shows again that the cutoff harmonics optimize for a different two-color phase than the plateau harmonics, similar to the observation and explanation of Fig. 4f.

We also observe that the divergence-resolved profile of each harmonic order in Fig. 4i still shows a substructure of two-color phases (colors) at which the yield optimizes. For example the harmonic at 28.5 eV shows orange wings in the vertical direction, and a green center. But in contrast to the other polarization angles, this trend is not as clear and uniform among all harmonics simultaneously. This is because for parallel polarizations, the two-color delay also acts as a gating mechanism in the ionization step, such that the yield of each harmonic will be optimized at a different two-color delay, corresponding to different colors for each harmonic in Fig. 4i. In addition, since the short and long trajectories have different ionization times, color contrast within a harmonic is still visible. This is in agreement with Fig. 2, in which we observed that also in the parallel two-color configuration there is a selection of long and short trajectories due to the changing ionization gate as function of two-color delay, albeit less pronounced than for perpendicular polarizations.

We now analyze the beam profiles in two-color HHG in more detail and systematically compare experimental and simulated results.

**Figure 5:**
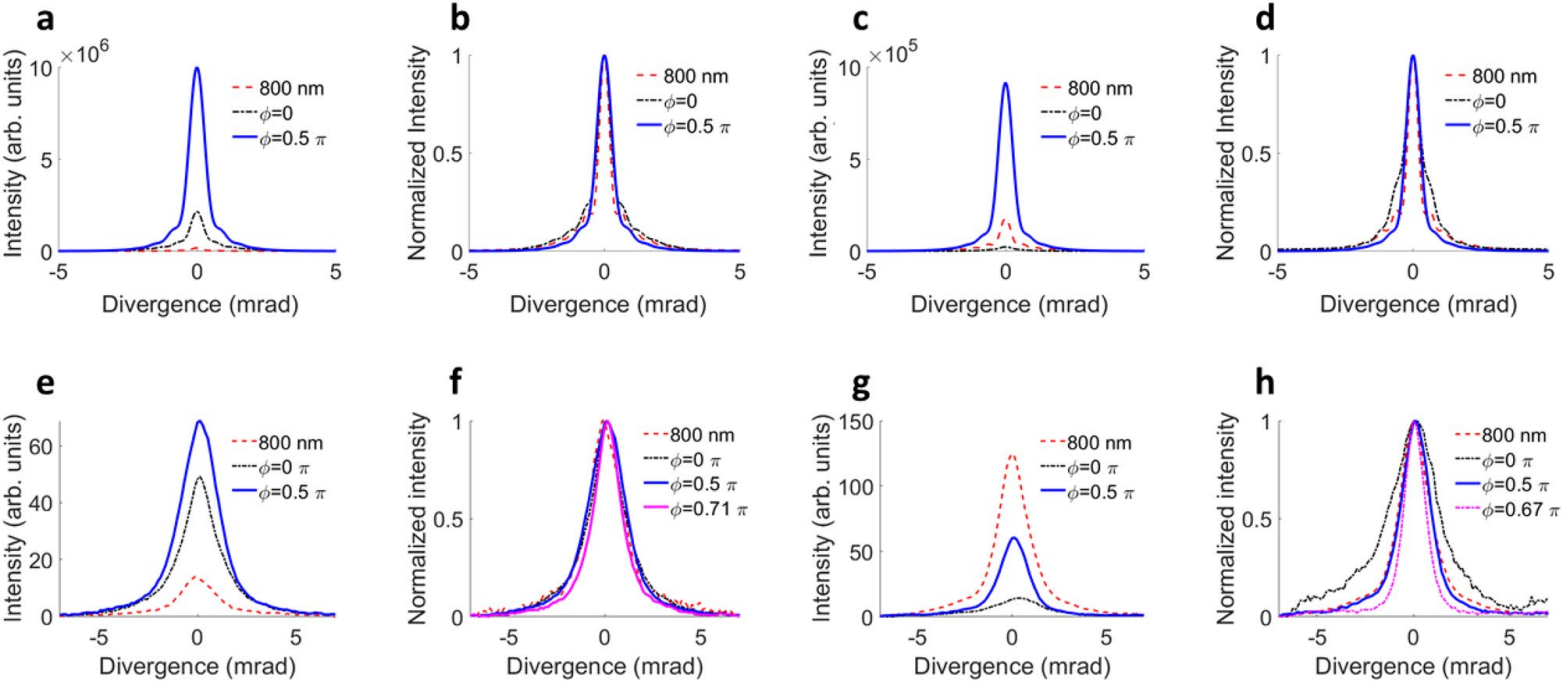
Simulated and experimental beam profiles: (**a**) Simulated parallel two-color HHG, yield comparison for 0 (black) and $0.5\pi$ rad (blue) phase delay and 800 nm only (red). (**b**) Simulated parallel two-color HHG, divergence comparison. (**c**) Simulated perpendicular two-color HHG, yield comparison. (**d**) Simulated perpendicular two-color HHG, divergence comparison. (**e**–**h**), experimental beam profiles, corresponding to (**a**–**d**).

The single-atom response of the medium is simulated for a thin slab of gas, around the focal plane. The harmonic field distribution is subsequently propagated to the far-field via Fraunhofer diffraction. More details can be found in the “Methods” section. The top row of Fig. 5 shows simulated, spectrally-integrated profiles.

For the two extreme cases, namely parallel and perpendicular polarizations, we compare the total yield (Fig. 5a,c) and the shape of the beam profiles after normalization (Fig. 5b,d). This is done for two phases, 0 and 0.5 $\pi$ rad (black dash-dotted and solid blue lines, respectively), which correspond to phases that optimize long and short trajectories for the perpendicular configuration, respectively. The yield and beam profile of the monochromatic configuration is also shown as a dashed red line, for comparison.

The parallel two-color scheme leads to a much higher yield compared to the one-color case, for both phase delays, as shown in Fig. 5a. Especially for a phase delay of $0.5\pi$ rad, the yield is greatly enhanced. For the same phase delay, the shape of the beam profile is also slightly less divergent compared to the one-color configuration as shown in the normalized profiles in Fig. 5b.

In the perpendicular configuration, a phase delay of $0.5\pi$ rad leads to the highest yield (Fig. 5c), which is also enhanced compared to the one-color case. Additionally, a two-color phase of $0.5\pi$ leads to an overall narrower beam shape (Fig. 5d).

The simulations are compared with the experimental results in the bottom row of Fig. 5, for the same two-color phases of 0 and $0.5\pi$ rad, and for the same spectral region (H13–H25). The total yield for the parallel configuration, shown in Fig. 5e, is in excellent agreement with the simulated data (panel a). Both delays lead to a considerably higher yield than in the monochromatic case, and the beam profiles show some phase-dependent modulation as well: there exists a phase (0.71 $\pi$ rad) that minimizes the overall divergence of the harmonic beam, as shown in Fig. 5f, which is explained by trajectory selection through gating the ionization times in the earlier sections of this article.

In the perpendicular case, the two-color phase has a large influence on the yield of the emission. In our experiments, the monochromatic case leads to a higher yield (Fig. 5g) than both two-color phases, in contrast with our simulations (Fig. 5c) and our previous findings^27^, where we showed that perpendicular polarized two-color HHG can improve the yield as well. We attribute this discrepancy to the necessary introduction of a half-wave plate for 800 nm into the common beam path of 800 nm and 400 nm for adjusting the relative polarization between both colors in the present article. This waveplate was identified to introduce some ellipticity to the 400 nm beam, lowering the total yield that could be achieved with a cleaner overall polarization state in the absence of a waveplate^27^.

The perpendicular two-color configuration has dramatic effects on the divergence (Fig. 5h). The beam profiles for a phase of 0 and $0.5\pi$ rad show a similar trend as the simulation. For the phase of $0.5\pi$ rad the beam profile becomes more narrow than the monochromatic case. Conversely, there is a two-color phase (0 rad) that makes the contribution of the long trajectories dominant at large propagation angles, thus increasing the divergence. In addition, we show a two-color phase that minimizes the long trajectories (0.67 $\pi$ rad), where their contribution vanishes almost entirely, so that the overall beam profile approaches a perfect mono-Gaussian distribution.

**Figure 6:**
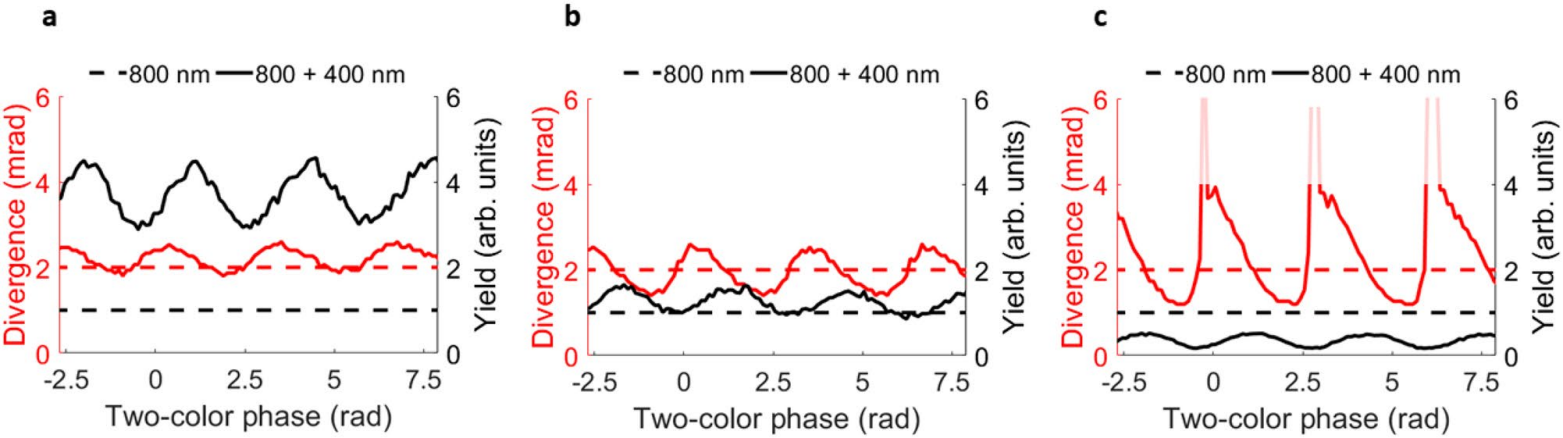
Divergence and yield, for different relative polarizations between SH and fundamental, as a function of two-color phase. (**a**) Parallel polarizations, leading to similar divergence as 800 nm generation, and much higher yield compared to 800 nm generation. (**b**) Two-color configuration, with 45° relative polarizations, showing improved divergence, and improved yield compared to 800 nm only. (**c**) Perpendicular two-color configuration, showing improved divergence compared to 800 nm only. The faded out areas correspond to profiles that became so broadened that a reliable extraction of the divergence angle at an intensity of $\text {1/e}^2$ became unfeasible.

The results in Fig. 5 have shown the strong influence of two-color HHG on the yield and beam profile/divergence. We now analyze further which two-color delays optimize divergence and yield, respectively, to see if both quantities can be optimized simultaneously. In Fig. 6, we track the total yield (black line) of the harmonics for each phase delay, which we obtain by summing the total signal of the detected harmonics. The total yield is normalized to the total 800 nm yield, to eliminate detection efficiencies depending on polarization. Second, we determine at which divergence angle the intensity of the beam profile reaches a $\text {1/e}^2$ value, which we show by the red line. We compare these values with the values obtained from HHG with the fundamental only, which are shown by the dashed lines. We compare the divergence of the two-color data for all polarization configurations to the perpendicular value.

The first remarkable observation is that for any two-color delay in the parallel configuration, the yield of the harmonics is higher than for the one-color configuration, see Fig. 6a. Second, tracking the divergence observable, we can see that there is a dependence on the two-color delay. The beam profile becomes narrower than in the one-color case for most of two-color delays. Interestingly, the yield and divergence do not optimize at the same two-color delays. This is expected, as the yield will optimize for maximizing both short and long trajectories, whereas the divergence changes when either selecting the short or the long trajectory.

For an intermediate polarization, the yield in the two-color case is again still higher for any two-color phase than for the one-color configuration, illustrated in Fig. 6b. Also, we see an improved divergence, depending on the two-color delay.

For a perpendicular two-color configuration, we can see that the divergence curve shows an even larger contrast than for the other polarizations, as shown in Fig. 6c. This confirms the significant control over trajectories in this configuration. The overall yield is lower than for the one-color configuration, which we discussed before in the text around Fig. 5.

All in all, the results in Fig. 6 show that for an appropriately chosen two-color phase, two-color HHG can increase the yield and decrease the divergence and thus improve both quantities. However, the phase where divergence and yield optimize is not identical. The relative polarization between SH and fundamental allows an experimentalist to choose between perfecting either the divergence (perpendicular polarizations) or the yield (parallel polarizations).

**Figure 7:**
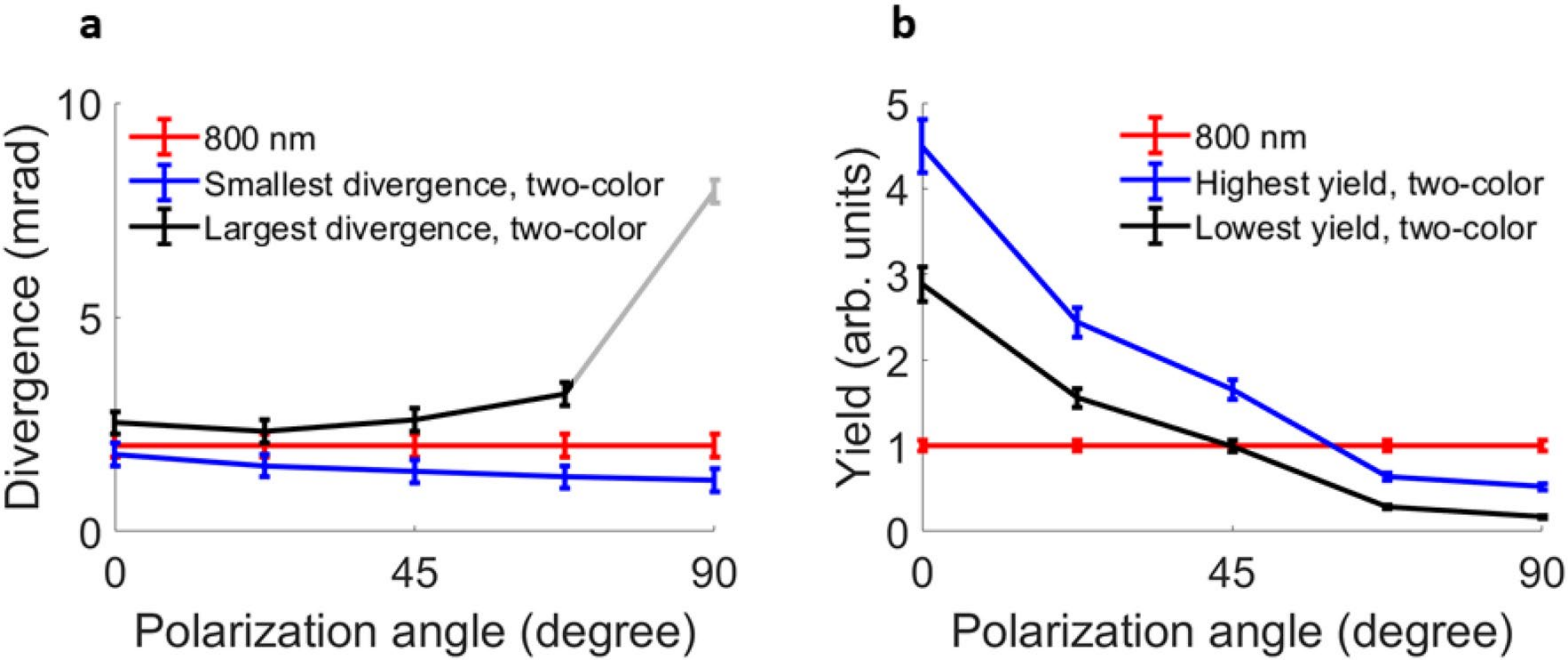
Summary of divergence and yield in two-color HHG. (**a**) The blue line shows results for two-color phases, which minimize the divergence, as a function of the polarization angle between both colors in the two-color field. This is compared to results obtained with a two-color phase where the divergence is broadest (black), and to the divergence of HHG with the fundamental only (red). The last data point for largest divergence (black) at 90° polarization angle is faded out, as it corresponds to a very divergent beam profile where an accurate divergence extraction becomes difficult, similar to the results in Fig. 6c. (**b**) Highest (blue) total yield for two-color HHG, compared to the lowest (black) total yield, and the 800 nm only configuration (red), for five different polarizations.

We have summarized our results, for 5 relative polarization angles in Fig. 7. The overall yield is the highest in the parallel case. For 67.5° and larger angles, the yield becomes lower than in the one-color configuration. Even for these angles we do expect that two-color HHG can still be made more efficient than one-color HHG as shown in Ref.^27^, and the discrepancy was explained in the previous section by the necessary introduction of a waveplate. Divergence control is the clearest in the perpendicular two-color configuration, as the contrast between the narrowest and broadest divergence at $\text {1/e}^2$ is the largest at this polarization angle. Still, for all polarization angles, divergence control is visible.

We want to stress the importance of the divergence and yield control for intermediate polarization configurations with respect to the polarization state of the generated high-harmonics. The divergence control demonstrates that the electron trajectories are modified depending on the two-color phase. As mentioned before, and shown in^44–46^, the lateral component in cross-polarized two-color HHG will lead to a classical angular momentum of the electrons relative to the atom. If both half-cycles are symmetric, which is the case for perpendicular cross-polarized two-color fields, the angular momentum obtained in each half-cycle will cancel out with the adjacent symmetric cycle and the harmonics will be linearly polarized. For intermediate cross-polarized configurations, adjacent half-cycles are not symmetric, as described around Fig. 4f, such that there is an effective angular momentum for the electrons which will be translated into elliptically polarized harmonics. Although we have not measured the ellipticity explicitly, the agreement between the SFA simulations and experimental data throughout our paper gives confidence that the harmonics are indeed elliptically polarized. The generation of elliptically and circularly polarized extreme-ultraviolet pulses with two-color fields provides opportunities for circular dichroism in the extreme-ultraviolet range^50^.

### Conclusion

In conclusion, we have conducted an extensive parameter study to identify the effects of the relative polarization between the two colors in two-color high-harmonic generation. We have compared the divergence and the yield with the one-color configuration. A parallel polarization of fundamental and SH in the two-color field leads to the highest yield enhancement. A perpendicular configuration leads to the best divergence control. The simulations, that take into account the single-atom response and phase matching in two-color fields, quantitatively reproduce the experimental beam profiles as a function of two-color phase and relative polarization between fundamental and SH. For a qualitative understanding, we find that all observations can be rationalized by the single-atom response in terms of trajectory selection and gating the tunnel ionization window in two-color laser fields.

## Methods

### Experimental setup

The experimental setup consists of the laser and optics, and the high-harmonic generation and detection chamber. A Ti:Sapphire (Coherent Astrella) laser generates 35-fs pulses at 800 nm central wavelength and 1 kHz repetition rate. The pulses are frequency doubled to 400 nm, using a 0.2 mm BBO (Castec). The power of the SH pulses was kept constant at 200 mW. The total power of the two-color pulses was 1.6 W. Assuming similar pulse lengths, similar focal spot sizes and perfect temporal synchronization, this leads to an SH intensity fraction of about 14%. The delay between fundamental and SH is compensated using two calcite plates. By motorizing the rotation angle of one of the calcite plates, sub-cycle control over the delay between the two colors was possible. To control the relative polarization, a half-wave plate for the 800 nm pulses was used, which acts roughly as a zero-wave plate for the 400 nm. Therefore only the polarization of the 800 nm pulses was changed, while keeping the 400 nm polarization constant. The two-color pulses are then focused inside an effusive Argon gas jet, using a 75 cm focal length aluminum spherical mirror. The gas jet was positioned using a 3D translational stage just before the focus. This configuration intrinsically minimizes the divergence of the short trajectory contributions^32^, because the dipole phase and phase front curvature of the fundamental have opposite signs and thus partially cancel each other. Simultaneously, the more divergent long trajectory contributions are efficiently phase matched with the gas before focus^31,51^, but can be suppressed through the action of the SH as shown in this article and in Ref.^27^.

The generated harmonics are spectrally dispersed with a concave abberation-corrected flat-field grating, and are detected using a double-stack microchannel plate backed with a phosphor screen. The harmonics freely propagate in the vertical direction, while being focused in the horizontal direction by the grating. The phosphor screen is imaged used a CMOS camera. In order to compare two-color HHG with HHG driven by monochromatic 800 nm pulses, the BBO was taken out, and the intensity of the monochromatic and two-color pulses was matched, to obtain similar cutoffs and therefore a fair comparison between the two generation schemes. We compare the two-color data with the one-color configuration for the spectral range of harmonic 13 to harmonic 25.

### Analysis of two-color delay scans

Figure 4c,f,i represent compact displays of entire scans of a divergence- and energy-resolved HHG spectrum as a function of the two-color phase. The nearly sinusoidal modulations of the HHG signal as a function of the two-color phase are Fourier transformed pixel-by-pixel. The frequency component with the highest amplitude of this Fourier transform then corresponds to the dominant oscillation frequency of the HHG signal as function of two-color phase. The amplitude of this frequency is represented as brightness in Fig. 4c,f,i, whereas the phase is represented as color. Hence HHG intensities that oscillate with different phases as function of two-color delay—such behavior is expected for long and short trajectories—are then directly visualized as color contrast in the figures. Thus this method provides a visual identification of long and short trajectory contributions to a divergence-resolved HHG spectrum.

The HHG intensity modulations as a function of two-color phase are periodic in $\pi$ rad, where a two-color phase delay of $\pi$ rad corresponds to a relative two-color delay of 0.67 fs.

### Simulations

The time-frequency plots in Fig. 2 were obtained by calculation of the two transverse components of the single-atom time-dependent dipole under the influence of the two-color laser field, using the strong-field approximation (SFA) integral with a hydrogenic transition matrix element^47^. Subsequently, for each time in the optical cycle a frequency analysis was performed by multiplying the time-dependent dipole using a Gaussian window function with an r.m.s. width of 0.4 $\omega$ centered around the time under investigation, Fourier transforming, and subtracting the ionization potential. A total intensity of $10^{14}$ $\text {W/cm}^2$ with a relative contribution of 20% second harmonic light was assumed.

The beam profiles shown in Fig. 5 were obtained by using the single-atom time-dependent dipole data described above as input to a propagation code based on the slowly varying envelope and paraxial approximations^48^, which coherently adds the emitted contributions of each volume element of an extended gas medium while taking diffraction and dispersion effects into account. The time- and position dependent amplitude and phase of both laser fields where calculated by propagation through the gas medium while keeping track of ionization-induced refractive index changes. The resulting XUV beam profile in the far field was obtained by a Hankel transform. In these calculations, we assumed Gaussian laser beams focused to a $1/e^2$ waist radius of 35 $\upmu \text {m}$, with pulse energies of 720 $\upmu \text {J}$ and 180 $\upmu \text {J}$ for the fundamental and second harmonic respectively, and a Gaussian temporal pulse shape with a FWHM length of 80 fs for both. A flattop gas target was assumed with a length of 220 $\upmu \text {m}$ and a pressure of 100 mbar argon, placed at 4 mm upstream of the focus.

## Data Availability

The data that support the plots within this paper and other findings of this study are available from P.M.K. upon reasonable request.
